# Changes in mental health in compliers and non-compliers with physical activity recommendations in patients with stress-related exhaustion

**DOI:** 10.1186/s12888-015-0642-3

**Published:** 2015-11-04

**Authors:** Agneta Lindegård, Ingibjörg H. Jonsdottir, Mats Börjesson, Magnus Lindwall, Markus Gerber

**Affiliations:** Institute of Stress Medicine, Carl Skottsbergs gata 22B, Gothenburg, SE-41319 Sweden; Department of Food and Nutrition, and Sport Science, University of Gothenburg, PO Box 300, Gothenburg, SE-40530 Sweden; The Swedish School of Sport and Health Sciences, University of Stockholm, Lidingövägen 1, Stockholm 1, SE-11433 Sweden; Department of Cardiology, Karolinska University Hospital, Stockholm, SE-17176 Sweden; Department of Psychology, University of Gothenburg, PO Box 500, Gothenburg, SE-40530 Sweden; Department of Sport, Exercise and Health, University of Basel, Birsstrasse 320B, Basel, CH-4052 Switzerland

**Keywords:** Anxiety, Burnout, Compliance, Depression, Physical activity, Stress-related exhaustion

## Abstract

**Background:**

There is a lack of research regarding the long-lasting effects of a more physically active lifestyle in patients with mental disorders. In the present study, clinical data were analysed to examine if initially physically inactive patients, clinically diagnosed with stress-related exhaustion, taking part in 12-month multimodal treatment (MMT), differ at the 18-month follow-up regarding mental health, depending on whether they did or did not comply with the physical activity (PA) recommendations resembling those of the American College of Sports Medicine.

**Methods:**

The study population consisted of 69 patients (65 % women) who were referred to a stress clinic due to stress-related exhaustion. All patients received MMT. A major goal was to increase patients’ PA levels. The patients received general comprehensive instructions including personal advice regarding the positive effects of PA on mental health and could self-select for an 18-week coached exercise program. Changes in mental health symptoms over an 18-month period were compared between non-compliers (*n* = 26), mild compliers (*n* = 22) and strong compliers (*n* = 21) with the PA recommendations included in the MMT.

**Results:**

Non-compliers, mild and strong compliers did not differ regarding burnout, depression and anxiety at baseline. Although substantial improvements occurred in all groups, mild and strong compliers reported significantly lower burnout and depression levels at the 18-month follow-up than the non-complying group (*p* < .05). The general pattern of findings was corroborated, if standard cut-off criteria for clinical burnout were used.

**Conclusions:**

Compliance with PA recommendations is associated with decreased levels of burnout and depression in patients with stress-related exhaustion. Thus, the promotion of a more active lifestyle among patients with stress-related exhaustion should be implemented as a part of MMT, to achieve a more sustainable decrease of symptoms of burnout and depression.

**Trial registration:**

This is not a clinical trial.

## Background

There is increasing evidence for the beneficial effects of physical activity (PA) in patients with mental disorders [1–4]. For instance, Knöchel et al. [5] showed that PA is an effective treatment, influencing both biological and psychological measures among patients with psychiatric disorders, leading to improved metabolic responses, increased quality of life and reduced psychopathological symptoms of cognitive failure.

So far, most research has focused on the effects of PA on depression and anxiety. With regards to depression, researchers have carried out several narrative reviews [6, 7]. In addition, several meta-analyses have been performed on data from randomized controlled trials [8–10]. Based on these meta-analyses, researchers reported overall effect sizes varying between −0.80 and −1.39, showing that regular PA contributes to reduced symptoms of depression. When examining moderating factors, Rethorst et al. [10] found that the effects were stronger in clinically depressed participants (ES = −1.03) compared to non-clinically depressed participants (ES = −0.59). Furthermore, Mead et al. [11], Krogh et al. [12] and Josefsson et al. [1] consistently showed that the effect sizes decreased if only studies with high methodological quality were included.

Several meta-analyses also exist with regards to the anxiolytic effects of PA. Wipfli et al. [13] showed that anxiety decreased more in the exercise groups than in the control groups with an overall effect size of -0.48. Furthermore, a moderator analysis revealed that the impact of exercise was similar in clinical (ES = −0.52) and non-clinical populations (ES = −0.40). Additionally, Rebar et al. [14] compared the effects of PA on depression and anxiety in non-clinical adult populations. Their meta-analysis showed that the effect was moderate for depression (ES = −0.50) and small for anxiety (ES = −0.38). A similar conclusion was reached by Conn et al. [15, 16] who found a larger overall effect size for depression (ES = 0.39 to 0.52) than for anxiety (ES = 0.22).

Rosenbaum et al. [3] recently conducted a meta-analysis to examine the impact of PA interventions for people with mental illness. In their study, they included participants with a broad range of psychiatric conditions such as major depressive disorder, schizophrenia, postnatal depression and first episode of psychosis. Again, this meta-analysis showed a large effect size on depressive symptoms (ES = −0.80). Furthermore, trial interventions that met the guidelines of the American College of Sports Medicine (ACSM) for aerobic exercise (ES = −0.94) did not differ significantly from those that did not meet these standards (ES = −0.61).

Taken together, we learn from these meta-analyses that numerous studies have been carried out to test the role of PA in the prevention and therapy of depression and anxiety. Typically, these meta-analyses are based on randomized controlled trials, which compare exercise with no treatment, an alternative treatment or a placebo condition. Undoubtedly, the great merit of these studies is that they have provided strong evidence in favour of the positive impact of PA on mental disorders, including major depression and anxiety. However, the vast majority of these trials have assessed short-term effects of exercise (e.g. [17–20]) and in none of the meta-analyses compliance has been studied as a moderating factor. However, measures of compliance are greatly warranted because the effects of an intervention strongly depend on participants’ compliance. In other words, an intervention may be perfectly designed, but high compliance is still very important for its effectiveness [21].

To date, we have only identified one relevant study in which long-term effects of exercise on depression were investigated [22]. In this study, the authors examined the 1-year follow-up of a 4-month, controlled clinical trial of exercise and antidepressant medication in patients with major depressive disorder. The findings show that while patients receiving exercise achieved similar benefits to those receiving antidepressants (compared to placebo controls) at the end of the intervention period, neither initial treatment group assignment nor antidepressant medication used during the follow-up period predicted the depression outcomes at 1 year. However, and most importantly, regular exercise during the follow-up period predicted both the Hamilton Depression Rating Scale scores and MDD diagnosis at 1-year follow-up.

In light of the current state of research, we conclude that little is known about the sustainability of the effects of exercise programs after the end of intervention trials. Considering the promising findings reported by Hoffman et al. [22], more research concerning patients’ exercise participation following the completion of therapeutic treatment seems warranted. Furthermore, very few studies have focused on the potential of exercise as a treatment for patients suffering from severe burnout symptoms [23, 24]. A pilot study with 12 male burnout patients showed that a 12-week aerobic exercise program has the potential to decrease participants’ levels of perceived stress, burnout symptoms, and to trigger improvements in their executive function [23, 25]; Bretland and Thorsteinsson [24] also revealed that 4 weeks of cardiovascular or resistance exercise result in decreased emotional exhaustion and increased personal accomplishment in a randomized controlled trial with 49 previously inactive volunteers. Moreover, to the best of our knowledge, no studies have examined whether it is possible to accomplish improvements in mental health by promoting PA as a part of MMT in a clinical setting. From a practical point of view, such studies are much needed because they have a high degree of external validity and provide findings that are more generalizable than those obtained from randomised controlled studies [26].

In the present study, patients with stress-related mental health problems are assessed. Stress-related disorders are an important health issue in the working population because they are one of the primary and fastest growing causes of long-term sick leave [27]. Depression and anxiety and clinical burnout are some of the most common mental consequences of chronic stress-exposure. In Sweden, exhaustion disorder (ED) is used in clinical practice to diagnose patients with symptoms of clinical burnout [28]. When depression and/or anxiety are present, which is common [29], ED should be set as a co-morbid condition. ED was registered within the IDC-10 system for diagnoses in 2005 (F43.8). The aim was to better define the patient category with severe mental health problems, caused by long-lasting identifiable stressors, eventually contributing to better treatment. ED causes markedly reduced physical and mental energy, a great variety of physical symptoms and a pronounced decline in cognitive functions, over a sustained period of time [29–31]. Thus, effective treatment strategies are warranted.

Recently, we have shown that it is possible to significantly increase PA levels over time in patients diagnosed with ED, by using the concept of graded PA. This means gradually increasing the amount of PA in a time-contingent way with the ultimate goal of developing a more physically active lifestyle [32]. This is an important finding given that physical inactivity is more common among individuals with mental disorders compared to the general population [33, 34].

Hence, the aim of this study was to expand previous research by investigating whether initially physically inactive patients diagnosed with ED differ at the 6-month, 12-month and 18-month follow-up regarding burnout (as a primary outcome) and depressive symptoms and anxiety symptoms (as secondary outcomes) depending on whether they (mildly or strongly) complied or did not comply with the PA recommendations resembling those of the American College of Sports Medicine (ACSM) [35, 36]. The PA recommendation was included as a part of a multimodal treatment (MMT) program, which is described in more detail in the method section.

## Methods

### Participants and screening procedure

**Fig. 1:**
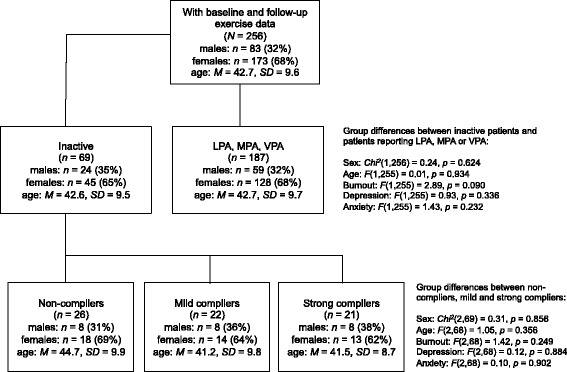
Differences between patients who were inactive versus active at baseline and differences between compliance groups regarding age, sex, burnout, depression and anxiety

Clinical data from patients referred to and treated at the Institute of Stress Medicine, Gothenburg, Sweden were used in this study. All participants were outpatients, being referred from primary health care centres, occupational health service centres or general practitioners because of stress-related exhaustion. Only patients fulfilling the diagnostic criteria for ED [28] and on on-going sick leave for less than 6 months entered an individualized MMT program at the clinic. Patients diagnosed with alcohol abuse, serious psychiatric diagnoses other than depression and anxiety, chronic fatigue syndrome or fibromyalgia, were not admitted to the clinic and therefore not eligible to enter this study. Data regarding PA at the 18-month follow-up was available for 256 patients. Among these, 69 identified themselves as physically inactive at baseline (27 %), 125 were engaged in light PA (49 %), 58 reported moderate PA (23 %), and four patients engaged in vigorous PA (1 %) (Fig. 1). PA was assessed with the Saltin-Grimby Physical Activity Level Scale (SGPALS; see below for more details). Only data from patients characterising themselves as physically inactive at baseline were included in this study (*n* = 69; 45 women, 24 men; *M*_age_ = 42.6 years, *SD* = 1.4). No differences were found with regard to sex, age, burnout, depression and anxiety between patients who were physically inactive and those who reported themselves to be active at baseline (see Fig. 1).

### Data assessment procedures and ethical considerations

A postal questionnaire, including a 1-item question regarding the patients’ PA level was sent to all patients shortly before the first visit to the medical doctor. The information collected served as the baseline measurements for the group comparison. In connection with the follow-up visits after 6, 12, and 18 months, the patients completed the follow-up questionnaires measuring PA and symptoms of mental health.

This study was approved by the regional ethical review board in Gothenburg, Sweden (Approval Nr. 243-05) and was conducted in accordance with the ethical principles described in the Declaration of Helsinki. Only patients who consented to the use of their clinical data for research purposes were included.

### Assessment of baseline physical activity

Baseline PA was assessed with the SGPALS [37], which distinguishes between individuals who are mostly physically inactive (level 1), who engage in light PA (e.g. gardening or walking or bicycling to work) at least 2 hours a week (level 2), who report at least 2 hours per week of moderate PA (e.g. aerobics, dancing, swimming, soccer, heavy gardening: level 3), or who engage at least 5 hours but several times per week in vigorous activity (level 4). Participants reported their PA for the last 3 months. This instrument discriminates between physically inactive and active individuals regarding their maximal oxygen uptake [38], has been validated against biological measures [39, 40], and against risks for morbidity and premature death [41–43].

### Assessment of follow-up physical activity

At follow-up, participants were asked to report the amount of weekly PA (frequency, intensity and duration) to examine whether patients comply with the ACSM’s guidelines for cardiorespiratory exercise [35, 44]. The ACSM’s guidelines for cardiorespiratory exercise recommend that adults should get at least 150 min of moderate-intensity exercise per week. Besides that, exercise recommendations can be met through 30–60 min of moderate-intensity exercise (5 days per week) or 20–60 min of vigorous-intensity exercise (3 days per week). Nevertheless, people unable to meet these minimums can still benefit from some activity.

First, patients were asked to report how often they had exercised during the last 3 months. Response options were a) not at all b) now and then c) 1–2 times, d) 3–4 times, and e) ≥5 times a week. To assess exercise intensity, patients were asked to report how hard they normally exercised during the last 3 months with response options from 0 (I do not get out of breath) to 4 (I feel completely exhausted). To assess exercise duration, patients indicated how many minutes they engaged in activity at this level, with the following response options: 1 = less than 20 min, 2 = 20–30 min, 3 = 30–60 min, 4 = more than 60 min.

Three groups of initially physically inactive patients were compared with reference to the ACSM’s guidelines: The first group consisted of non-compliers (*n* = 26) who were still physically inactive at 18-month follow-up and thus did not accomplish the ACSM’s guidelines. The second group was composed of mild compliers (*n* = 22) who reported some PA (at least once per week), but did not meet the ACSM’s recommendations for cardiorespiratory exercise. The final group was composed of strong compliers (*n* = 21) who adhered with the ACSM’s standards. Patients in this group engaged (a) at least five times per week for at least 30 min in moderate exercise or (b) at least three times per week for at least 20 min in vigorous exercise.

### Burnout symptoms

Burnout symptoms were measured with the 22-item Shirom-Melamed Burnout Questionnaire (SMBQ) [45]. Sample items are: ‘I feel no energy for going to work in the morning.’ ‘I feel I am not thinking clearly.’ Response options are anchored on a 7-point Likert scale from one (*almost never*) to seven (*almost always*). Mean scores were calculated to generate an overall index. The SMBQ has proven to be a valid and reliable instrument in prior research [46–48]. Scores ≥4.47 were regarded as a clinically relevant [47]. The Cronbach’s alpha was .92 in the present sample.

### Anxiety and depression

The 14-item Hospital Anxiety and Depression (HAD) scale was used to measure the participants’ symptoms of depression and anxiety [49]. Subscale scores are based on participants’ answers to one of four response options on a 4-point Likert-scale (ranging from 0 to 3) regarding mood changes that may occur during the course of depression (e.g., ‘I still enjoy the things I used to enjoy.’) or anxiety (e.g., ‘Worrying thoughts go through my mind.’). The HAD-instrument has been shown to be a valid and reliable instrument in previous studies [50–53]. The sum score was calculated to obtain two overall indices for depression and anxiety. Subscale scores >10 were considered clinical, as previously described [49, 50]. In the present study, the Cronbach’s alphas for depression and anxiety were .85, respectively.

### Multimodal treatment

All patients obtained MMT with similar components. The composition of the program was tailored according to each participant’s individual needs. Thus, only patients with sleep disturbances were offered therapies to improve sleep, and only patients with depression where offered antidepressants. The frequency and duration of visits to different health personal was similar for all patients (on average, patients had two consultations lasting 1.5 h and 10 consultations lasting 30 min), and all patients were given background information on the causes and consequences of chronic stress during a 2-h lecture. Additionally, an 8-week group stress management program was offered to all patients. Because there is very little variation in the components of the MMT with regard to attendance, no clear groups with distinct patterns of attendance and/or compliance could be identified. Usually consultations with the physician took place at regular intervals of 4 to 6 weeks. During the visits, the physician and the patient frequently discussed lifestyle-related topics. Some patients with serious self-rated sleep problems were invited to take part in cognitive behavioural group therapy, focusing on different sleep disturbances, and/or were advised to visit a psychologist for individual psychotherapy. Antidepressants were offered or adjusted where it seemed appropriate. The MMT also included the possibility for employers, working colleagues and relatives to attend a 2-h lecture about stress-related mental disorders and the consequences on the individual and organisational level (e.g., possible effects of stress-related disorders on work performance and work ability).

A special focus was placed on PA counselling to encourage patients to start or increase their PA level as a part of the treatment. All patients received 1.5 h of comprehensive information on the effects of regular PA on stress-related exhaustion, from a specialised physiotherapist. The goal was to establish an individually tailored dose of PA, with respect to stress-related exhaustion as well as possible co-morbidity. Moreover, all patients had the opportunity to self-select their participation in an 18-week coached group-exercise program comprising Nordic walking for one hour and a light strength-training program performed at the clinic, once a week. Thus, the PA component differs from the other components of the MMT in the sense that PA was recommended to all patients, but that compliance with these recommendations was fully based on patients’ choice. As a result, the compliance with the PA recommendations 6 months after the end of the 12-months treatment differed considerably between patients, enabling an isolated examination of the importance of this factor.

## Statistical analyses

Chi^2^-tests and univariate analyses of variance (ANOVAs) were used to test baseline differences between non-compliers, mild and strong compliers. To test group differences at the different measurement occasions, ANOVAS were carried out with burnout, depression and anxiety as dependent variables. To examine Time x Group effects, repeated measures ANOVAs were calculated. Following Cohen [54], η^2^ values from .010 to < .059 are interpreted as small effects, from .059 to < .138 as medium effects, and from .138 as large effects. To test whether groups differed with regard to clinical levels of burnout, depression and anxiety, Chi^2^-tests were carried out separately for every outcome at baseline, 6, 12, and 18 months of follow-up. All statistical analyses were performed with SPSS 20 (IBM) for Mac.

## Results

### Descriptive statistics and baseline group differences

As shown in Fig. 1, there were no significant differences with respect to sex, age, burnout, depression and anxiety between non-compliers, mild and strong compliers with PA-recommendations. Furthermore, no significant differences between the groups were found with regard to the use of antidepressants, BMI, sick leave status, changes in occupation, job loss, retirement and physical comorbidity during the study period. At baseline, 93 % (*n* = 64) of the patients reported burnout symptoms above the clinical cut-off score (≥4.47), whereas 74 % (*n* = 51) exceeded the cut-off for clinical anxiety and 43 % (*n* = 29) scored above the cut-off for clinical depression (≥11). Furthermore, no differences could be seen between non-compliers, mild and strong compliers with respect to the ratio of patients who were involved in the coached exercise training or who only received general instructions as part of the MMT (non-compliers: 23 % in coached exercise, mild compliers: 46 %, strong compliers: 24 %), *Chi*^*2*^(2,69) = 3.44, *p* = .179.

### Follow-up group differences in mental health

With regard to the levels of burnout as the primary outcome, patients who complied mildly or strongly with the PA recommendations did not differ from non-compliers at baseline, at 6 and at 12 months of follow-up, but significant group differences emerged at the 18-month follow-up (Table 1).

**Table 1:** Group differences based on 1-way ANOVAs in burnout, depression and anxiety at baseline, 6, 12, and 18 months of follow-up

	Non-compliers (n = 26)		Mild compliers (n = 22)		Strong compliers (n = 21)					
Burnout	*M*	*SD*	*M*	*SD*	*M*	*SD*	*df*	*F*	*p*	η^2^
Baseline	5.49	0.91	5.69	0.59	5.26	0.86	2,65	1.42	0.249	0.043
6 months	4.55	0.97	4.10	1.38	4.22	1.17	2,63	0.85	0.431	0.027
12 months	3.81	1.02	3.56	1.60	3.64	1.29	2,65	0.81	0.806	0.007
18 months	3.97	1.43	2.97	1.19	3.09	1.15	2,65	4.36	0.017	0.121
Depression	*M*	*SD*	*M*	*SD*	*M*	*SD*	*df*	*F*	*p*	η^2^
Baseline	9.85	4.17	9.32	3.80	9.38	4.14	2,68	0.12	0.884	0.004
6 months	7.13	3.99	4.50	2.96	6.29	3.96	2,66	3.02	0.056	0.086
12 months	5.92	4.31	4.23	3.98	4.10	3.24	2,67	1.62	0.205	0.048
18 months	5.54	4.51	2.73	2.64	3.29	3.95	2,68	3.68	0.031	0.100
Anxiety	*M*	*SD*	*M*	*SD*	*M*	*SD*	*df*	*F*	*p*	η^2^
Baseline	12.58	4.05	12.14	3.26	12.52	3.23	2,68	0.10	0.902	0.003
6 months	9.29	3.13	7.27	4.07	8.05	3.29	2,66	1.94	0.152	0.057
12 months	7.23	3.56	5.73	3.37	5.65	3.01	2,67	1.71	0.190	0.050
18 months	6.77	3.66	4.73	3.27	4.65	3.39	2,67	2.90	0.062	0.082

Variations in number of cases dependent on missing values in different subscales

**Fig. 2:**
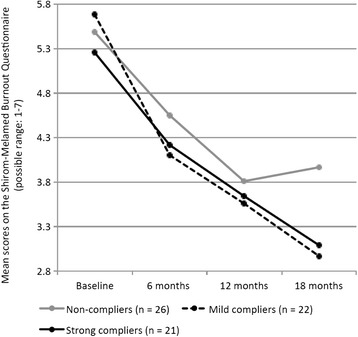
Changes in symptoms of burnout from baseline to 18-month follow-up across groups

The significant time effect found in the repeated measure ANOVA revealed that all groups decreased their level of burnout from baseline to the 18-month follow-up. Nevertheless, as illustrated in Fig. 2, burnout symptoms continued to decrease in mild and strong compliers, whereas an interruption of the initially positive trajectory was observed among non-compliers after the end of the 12-month MMT period. Moreover, the results of the repeated measure ANOVA (Table 2) demonstrate a significant time by group interaction, corroborating the notion that the burnout scores decreased significantly more in mild and strong compliers, compared to non-compliers. The η^2^ value of .131 pointed towards a moderate-to-strong effect.

**Table 2:** Overview of inferential statistics of the repeated measures ANOVAs separately for group (non-compliers, mild and strong compliers), and time (from baseline to 18-month follow-up)

	Group			Time			Group × Time		
	*df*	*F*	η^2^	*df*	*F*	η^2^	*df*	*F*	η^2^
Burnout	2,61	2.67	.080	1,61	161.14***	.725	2,61	4.59*	.131
Depression	2,66	1.90	.055	1,66	100.86***	.604	2,66	1.61	.047
Anxiety	2,65	1.61	.047	1,65	159.96***	.711	2,65	1.21	.036

Variations in number of cases dependent on missing values in different subscales

***p < .001

*p < .05

Regarding symptoms of depression, similar results occurred. At the 18-month follow-up, the group differences became statistically significant. Those patients who complied mildly or strongly with the ACSM’s PA guidelines experienced lower levels of depressive symptoms (Table 1). At the 18-months follow-up, the main effect for group explained 10 % of variance. Although the repeated measures ANOVA was unable to detect a significant Time x Group interaction (*p* = .081), the η^2^ value of .047 indicated that compliance with the PA recommendations was associated with a small effect (Table 2).

Concerning symptoms of anxiety, no significant group differences were found. Moreover, the repeated measures ANOVA did not detect a significant Time x Group effect (*p* = .304).

If the clinical cut-off level for burnout was used as an indicator of mental health, the data showed no significant differences at baseline, *Chi*^*2*^(2,69) = 2.54, *p* = .281, at the 6-month follow-up, *Chi*^*2*^(2,69) = 0.56, *p* = .754, and at the 12-month follow-up, *Chi*^*2*^(2,69) = 0.57, *p* = .750, while significant group differences occurred at the 18-month follow-up, *Chi*^*2*^(2,69) = 6.15, *p* = .046, showing that among the non-compliers a higher percentage of participants (*n* = 11; 42 %) exceeded the cut-off score for clinically relevant burnout compared to the mild compliers (*n* = 3; 15 %) and strong compliers (*n* = 3; 15 %). In contrast, no significant group differences were found at any time if clinical levels of depression and anxiety were examined (data not shown).

## Discussion

The main result of this study is that patients with exhaustion disorder, who complied mildly or strongly with the PA recommended as a part of the MMT program, showed larger and more sustained improvements in burnout during the follow-up period than non-compliers. Thus, patients who complied at least mildly in changing their PA habits continued to improve their mental health with respect to burnout over the 18-month follow-up period, which was not the case among non-compliers.

For burnout and depression, significant group differences between non-compliers and compliers with PA recommendations occurred only after the 18-month follow-up. This indicates that the emerging differences may indeed mirror an effect of the increased level of PA, rather than being effects of the MTT as a whole, since all other regular treatment components apart from follow-up appointments ended at the 12-month follow-up. Moreover, the non-compliers showed a slightly increased level of burnout between the 12-month and 18-month follow-up, compared to the two complier-groups who continued to show decreased symptoms. This strengthens the assumption regarding the explanation for the late appearance of the significant differences in symptoms of burnout.

At the 18-months follow-up significant group differences existed for burnout and depression, but not for anxiety. This observation can be explained by the fact that generally exercise has stronger anti-depressive than anxiolytic effects [13, 15, 16]. However, a significant Time × Group effect was only found for burnout, but not for depression and anxiety. Focusing on burnout as a primary outcome seemed justified because the data were assessed in a clinic specialized in the treatment of stress-related exhaustion disorder. Not surprisingly, therefore, nearly all patients reported clinical burnout levels (93 %), whereas a smaller portion reported clinical levels of depression (43 %) or anxiety (74 %). This is congruent with the notion that a certain overlap exists between burnout and depression, but that they are not identical constructs [55]. Thus, there seems to be a greater scope for improvement in symptoms of burnout among patients with stress-related exhaustion than for symptoms of depression and anxiety.

The present study demonstrated that patients who comply with the ACSM’s cardiorespiratory exercise recommendations show sustained improvements in burnout after the end of the MMT. This finding is in line with a recent study showing that an exercise dose corresponding to the one proposed by the ACSM has the potential to reduce burnout symptoms among males suffering from burnout [23]. Furthermore, vocational students meeting the ACSM’s recommendations reported lower burnout symptoms than peers who did not fulfill these standards [56, 57]. Interestingly, in the present study, a similar pattern of results was found for patients who did not accomplish the ACSM’s guidelines, but who reported at least one PA episode per week. Thus, they showed similar continued improvements after completion of the MMT as patients who met the ACSM’s guidelines. This contradicts the results described by Dunn et al. [19] who showed that aerobic exercise with a dose consistent with public health recommendations for PA resulted in a reduction of depressive symptoms after a 12-week intervention period, whereas a lower dose did not. On the other hand, our findings support previous research on the relationship between PA and burnout showing that the most significant differences in burnout are found between people who are completely inactive compared to those who engage at least in some PA [58, 59]. Furthermore, our results correspond well with the meta-analysis of Rosenbaum et al. [3] who found that the effects of intervention trials with people suffering from severe mental diseases were comparable independent of whether the dose of the exercise programs did or did not correspond with the ACSM’s standards. Furthermore, Hoffman et al. [22] showed a curvilinear relationship between self-reported exercise during follow-up and depressive status at 1 year, indicating that the most substantial improvements in depression outcomes occur before the 150 min/week criterion. Moreover, our findings accord well with ACSM’s position that people unable to meet the minimum standards can still benefit from some activity. Finally, as outlined by Gerber et al. [23], the negative relationship between exercise and burnout can be attributed to both psychological and physiological mechanisms. The fact that similar positive effects were found in the present study for participants who exercised 1–2 times per week compared to participants who met the ACSM’s exercise recommendations (at least 3–5 times active, depending on the intensity), indicates that the relationships might not exclusively be due to physiological mechanisms.

These findings and possible interpretations notwithstanding, we acknowledge that our self-report measure only provides a rough estimate of PA. In order to establish firm conclusions about dose and effect, further research is needed with instruments that allow a more precise assessment of PA participation or energy expenditure. Thus, while cautious interpretations are needed, we can propose the relationships identified in our study tentatively as hypotheses that need to be tested by further research using other questionnaires that allow a more fine-grained picture or objective assessments of PA.

Although there is strong evidence for the preventive effects of PA on mental disorders, including major depression [3, 58, 60–62], studies regarding the long-term effects of increased PA in a patient group with severe mental health problems, such as clinical burnout, are still very limited [22]. To the best of our knowledge, no studies have examined whether it is possible to accomplish improvements in mental health by promoting PA as a part of MMT in a clinical setting. From a practical point of view, such studies are much needed because they have a high degree of external validity and provide findings that are more generalizable than those obtained from ordinary randomised controlled studies [26]. Thus, the results showed that the increased levels of PA achieved in this patient group within the frames of a clinical setting with rather modest efforts [32], is paralleled by a decrease in symptoms of burnout, depression and anxiety within the same patient group. This indicates that promoting PA, as a part of MMT used in the therapy of stress-related mental disorders, is a worthwhile endeavour for practitioners to decrease the burden of disease among these patients.

Several methodological considerations are of interest to discuss. First, we only used self-report measures to assess the primary and secondary mental health outcomes. Although the instruments provide clinical cut-off scores, they do not allow a real clinical assessment. Second, we chose to measure PA with self-reports in this study. Although more objective PA assessment measures are available today, such as accelerometers, most studies investigating the link between PA level and health have used self-reports, showing their predictive validity [41, 58]. For patients with stress-related mental disorders, it has been elucidated previously that self-reported PA was significantly related to self-reported symptoms of burnout, depression and anxiety, whereas objectively measured aerobic fitness was not [59]. One important advantage of choosing self-reported PA corresponding to the ACSM’s recommendations was the plausible practical implication. Thus, the results of the present study can easily been implemented in clinical practice. Third, the rather small study group (*n* = 69) in this study can be considered a limitation, preventing us from performing separated analyses for men and women. Nevertheless, the distribution of men and women was similar across all groups. Moreover, in a similar patient population, no differences related to sex or age could be seen regarding course of mental illness over time [29]. Nevertheless, more studies are warranted with respect to possible differences in compliance and effects of lifestyle changes in general between men and women. Our study could be a first step towards filling the gap between randomised controlled studies and more clinically oriented studies, in order to facilitate the implementation of well-documented measures into everyday clinical practice. Fourth, one important methodological consideration is related to the MMT treatment which is by nature heterogeneous, adapted to the participant’s individual needs. The aim of this study was not to compare different components of the MMT, as this is neither possible nor useful due to the patient-tailored nature of MMT. Thus, no conclusion with regard to the effectiveness of individual components can be made (particularly as the groups showed similar improvements in burnout during the first 12 months). However, the PA component differs from the other components of the MMT in that PA was recommended to all patients, that compliance was fully based on patients’ choice, and that therefore, compliance varied considerably, thus enabling an isolated examination of this factor. Other components cannot be easily dissected from each other but the core features for each participant has been registered in the patients’ medical records. We could not detect any major difference between the groups with regard to the treatments components offered to the patients. The character of the registration of the MMT participation does not, however allow for statistical analysis, making it impossible to control for this factor in the analyses. Furthermore, while we did not find significant group differences with regard to age, sex, antidepressant use, sick leave status, physical comorbidities, and changes in occupation, job loss and retirement during the study period, we acknowledge that other (uncontrolled) factors may have had an impact on patients’ compliance with PA recommendations. Fifth, the present paper focused on patients that were initially physically inactive. However, this seemed justified because the primary goal of the PA counselling was to motivate inactive patients to adopt a more physically active lifestyle. A sixth methodological consideration is the classification of patients into groups based on compliance. As far as we know, this is the first study that attempts to define compliance based on the ACSM’s recommendation. The strong compliance category fully satisfies the recommendations of the ACSM, whereas the mild compliance category is somewhat arbitrary, including all patients who reported at least one weekly exercise episode, but did not accomplish the ACSM’s standards. Seventh, it should be noted that while the 18-month follow-up took place 6 months after the end of the MMT, the PA questionnaire referred to the last 3 months. Thus, it is likely that changes might have occurred during the first 3 months after completion of the MMT that were not assessed by the questionnaire. Nevertheless, Shephard [63] argued that “because of limitations in human memory, the reliability of information generally decreases with the length of the period surveyed”and that therefore “it is best to keep the reporting interval relatively short (no longer than 3 months)”(p. 199).

## Conclusions

Patients diagnosed with stress-related exhaustion, with initially inactive lifestyles, who managed to integrate at least one weekly exercise episode in their regular behaviour patterns, reported the largest improvements in burnout at follow-up. Therefore, we claim that a PA component should be implemented as a vital part of MMT programs among patients diagnosed with stress-related exhaustion.
